# A multimodal analytical method to simultaneously determine monoacetyldiacylglycerols, medium and long chain triglycerides in biological samples during routine lipidomics

**DOI:** 10.1186/s12944-022-01650-w

**Published:** 2022-05-10

**Authors:** Charles F. Manful, Thu H. Pham, Heather Spicer, Raymond H. Thomas

**Affiliations:** School of Science and the Environment/ Boreal Ecosystem Research Initiative, Grenfell Campus, Memorial University of Newfoundland, Corner Brook, NL A2H 5G4 Canada

**Keywords:** Thin layer chromatography, Triglyceride analysis, Acylated triglyceride, Liquid chromatography, Mass spectrometry, Functional lipids, *Eurosta solidaginis*

## Abstract

**Background:**

Monoacetyldiglycerides (MAcDG), are acetylated triglycerides (TG) and an emerging class of bioactive or functional lipid with promising nutritional, medical, and industrial applications. A major challenge exists when analyzing MAcDG from other subclasses of TG in biological matrices, limiting knowledge on their applications and metabolism.

**Methods:**

Herein a multimodal analytical method for resolution, identification, and quantitation of MAcDG in biological samples was demonstrated based on thin layer chromatography-flame ionization detection complimentary with C30-reversed phase liquid chromatography-high resolution accurate mass tandem mass spectrometry. This method was then applied to determine the MAcDG molecular species composition and quantity in *E. solidaginis* larvae. The statistical method for analysis of TG subclass composition and molecular species composition of *E. solidaginis* larvae was one-way analysis of variance (ANOVA).

**Results:**

The findings suggest that the proposed analytical method could simultaneously provide a fast, accurate, sensitive, high throughput analysis of MAcDG from other TG subclasses, including the fatty acids, isomers, and molecular species composition.

**Conclusion:**

This method would allow for MAcDG to be included during routine lipidomics analysis of biological samples and will have broad interests and applications in the scientific community in areas such as nutrition, climate change, medicine and biofuel innovations.

**Graphical abstract:**

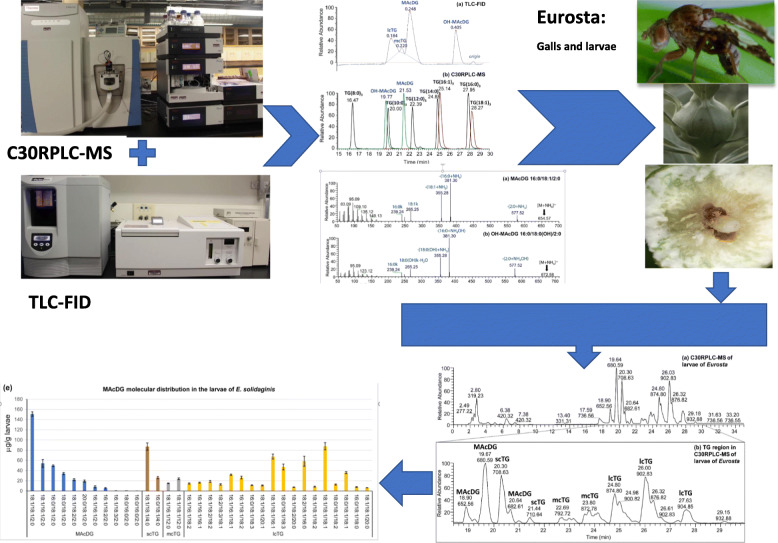

**Supplementary Information:**

The online version contains supplementary material available at 10.1186/s12944-022-01650-w.

## Introduction

Triglycerides (TG) are neutral lipids which provide primary energy reserves in living organisms [1]. Structurally, TGs are triesters composed of three fatty acids (FA) esterified at the stereospecifically numbered carbon 1 (*sn-1*), 2 (*sn-2*), and 3 (*sn-3*) positions of the glycerol backbone. They are involved in regulation of fatty acid metabolism and plasma levels of lipoproteins in human [2, 3]. TGs are divided into four subclasses based on their fatty acids. In this regard, monoacetyldiglycerides (MAcDG) are acetylated triglycerides having an acetate moiety (CH_3_COO-) at *sn-3* position and longer fatty acyl chains at *sn-1* and *sn-2* positions. Short chain triglycerides (scTG) contain at least one fatty acyl chain composed of 3–5 carbons. Medium chain triglycerides (mcTG) contain at least one fatty acyl chain composed of 6–12 carbons, while long chain triglycerides (lcTG) are composed of fatty acyl chains containing more than 12 carbon atoms [4]. lcTG are dietary sources of essential polyunsaturated fatty acids including docosahexaenoic acid (DHA-ω3), eicosapentaenoic acid (EPA-ω3), linolenic acid (ALA-ω3), arachidonic acid (ARA-ω6), and linoleic acid (LA-ω6). Several nutritional studies support consumption of these fatty acids and a reduction in risk factors for cardiovascular diseases, cancers, arthritis, diabetes, hypertension and neuropsychiatric disorders [5]. Furthermore, consumption of mcTG either in the diet or as supplements has been reported to be beneficial for reducing body fat and obesity [6], alcoholic liver diseases [7], and short bowel syndrome [8]. MAcDG have been reported to be effective in treating sepsis [9, 10], inflammations, cancers [11], arthritis [12], and asthma [13]. Furthermore, MAcDG is an emerging functional lipid in the dietary and health sector. It is also a useful adjuvant for improving the performance of biofuels under cold climatic conditions [14, 15].

As such, to fully exploit these benefits from MAcDG, there is a need for fast, accurate, precise and sensitive techniques to identify and quantify MAcDG from other TG subclasses in biological matrices. A major challenge exists in the scientific community with analyzing MAcDG from other subclasses of TG. In most routine lipidomics techniques, MAcDG is not resolved from the other subclasses of TG, and as such there is limited publications concerning their applications in the scientific community. Furthermore, there is current interest in the biofuel industry to use MAcDG as an adjuvant to resolve viscosity issues with biofuels when used in cold or northern climates. As a result, oil seed crops such as camelina is now successfully engineered to produce high levels of MAcDG for biofuel production and applications in cold climates [14, 15]. Thus, there is a need for the development of suitable analytical methods to permit the routine analysis of MAcDG to improve understanding of how this unique lipid function and resolve issues in health and industrial applications. The literature is replete with analytical methods for the analysis of TG in biological samples that could be useful for the analysis of MAcDG [16]. Among these, thin layer chromatography coupled with flame ionization detection (TLC-FID) provides cost effective, accurate, precise, and efficient method for routine quantification of TG subclass composition [17–19]. However, TLC-FID does not provide information on molecular species and fatty acid compositions, which is important for nutritional, medical, and industrial applications of TG. While mass spectrometry-based techniques including gas chromatography coupled to flame ionization detection (GC-FID) and gas chromatography coupled to mass spectrometry (GC-MS) provide higher sensitivity, accuracy, precision, linear range and throughput compared to TLC-FID for quantification of TG fatty acid composition, these methods are not applicable for quantification of intact molecular species composition of TG subclasses including MAcDG [19, 20]. Furthermore, acid/base hydrolysis and chemical derivatization steps required for sample preparation in GC-FID and GC-MS analysis of TG limits the general utility and scope of biological samples which can be analyzed by these methods [21]. Liquid chromatography-heated electrospray ionization tandem mass spectrometry (LC-HESI-MS/MS) allows identification and quantitation of intact TG molecular species, fatty acid, and subclass composition of biological samples in a single run [22, 23]. Furthermore, LC-HESI-MS provides higher accuracy, precision, and sensitivity compared to GC-MS and GC-FID, and does not require prior hydrolysis and derivatization steps for mass spectrometric analysis of TG [24].

Herein a multimodal analytical method for routine identification and quantitation of MAcDG as a subclass of TG in biological samples was described based on TLC-FID complimentary with C30-reversed phase liquid chromatography-tandem mass spectrometry (C30-RPLC-MS/MS). This method is further applied for quantification of the molecular species and fatty acid composition in biological samples using *E. solidaginis* larvae fat as an example. The proposed C30-RPLC-MS/MS method is the first to be applied for quantification of MAcDG molecular species, fatty acid, and subclass compositions in *E. solidaginis* larvae lipidome. Furthermore, the proposed TLC-FID method supplements the previous work [20] and demonstrates resolution of hydroxylated MAcDG (OH-MAcDG) in relation to MAcDG, mcTG, and lcTG by TLC-FID. The applicability of this proposed method is for the analysis of MAcDG to be potentially included during routine lipidomics assessments of biological samples.

## Materials and methods

### Standards and reagents

Triglyceride (TG) standard mix (Splash Lipidomix Standard- 17,811-1AMP) was obtained from Supelco (Oakville Ontario, Canada). Synthesized and purified MAcDG compounds, including 16:0/18:0/2:0, 16:0/9Z-18:1/2:0,16:0/9Z,12Z-18:2/2:0 and OH-MAcDG 16:0/18:0(OH)/2:0, were provided by Chemforce Laboratories (Edmonton, Canada). Calibration standard solutions (0–10 μg/mL) containing these standards were used as spiked internal standards to prepare standard curves to quantify TG in *E. solidaginis*.

### Collection of *E. solidaginis* larvae

*E. solidaginis* larvae were extracted from the galls of *Solidago* spp. plants into 2 mL Eppendorf centrifuge tubes and stored at − 80 °C. These plants were from old field habitats in London, Ontario, Canada (43°00′N, 81°15′W). Memorial University Animal Care provided ethics approval [20160041] for this study. All experiments complied with established guidelines and regulations.

### Lipid extraction from *E. solidaginis* larvae

Lipids were extracted in septuplicate (*N* = 7) from *E. solidaginis* larvae (85–170 mg) according to the Folch method using 2.4 mL chloroform/methanol (2:1, v/v) and 0.8 mL of 0.1% v/v butylated hydroxytoluene [25]. The resulting mixture was vortexed for 10 s and centrifuged at 10000×g for 10 min at − 4 °C. The organic layers were pooled into pre-weighed glass vials, and the solvent removed under dry nitrogen stream. Prior to TLC-FID analysis, the dried lipids were dissolved in chloroform (700–900 μg/mL).

### Thin layer chromatography-flame ionization detection (TLC-FID) analysis of MAcDG

A standard mixture containing MAcDG, mcTG, lcTG and OH-MAcDG standards was used for assessing the effectiveness of TLC-FID to resolve MAcDG from other classes of TG. The TLC-FID analysis of TG subclass composition of the complex standard mixture was conducted following the procedures described by Marshal et al. (2014) with minor modifications [20]. Briefly, 1.7 mL solution of the complex standard mixture in chloroform was resolved on chromarods (5 mm silica gel-coated quartz rod; Shell, Virginia, USA) using 70:30:0.5 benzene:chloroform:formic acid (v/v/v) for chromatographic separation. TLC-FID analysis was performed using an Iatroscan thin-layer chromatograph (Model MK-6 TLC-FID Analyzer; Shell, Virginia, USA) equipped with a flame ionization detector. The instrument was operated at the following settings: flow rate of 2 L min^− 1^ for atmospheric air, 160 mL min^− 1^ for hydrogen, and scanning speed of 3.0 cm s^− 1^ [20]. Identification of TG subclasses (MAcDG, mcTG, lcTG and OH-MAcDG) present in the complex standard mixture was achieved by comparing retention times and elution order of the individual standards (e.g. MAcDG) spotted on separate chromarods.

### MAcDG analysis by C30 reversed phase liquid chromatography-heated electrospray ionization high resolution accurate mass tandem mass spectrometry (C30-RPLC-HESI-HRAM-MS/MS) and application for the analysis of MAcDG in *E. solidaginis* fat

In this section, the application of C30-RPLC-HRAM-MS/MS was demonstrated for the analysis of MAcDG in a complex standard mixture and a biological sample (*E. solidaginis* fat) containing several different subclasses of TG (MAcDG, mcTG, ScTG, lcTG). LC–MS analysis of *E. solidaginis* fat followed the same procedures described in previous publications [26, 27]. Briefly, an Accucore C30 column (150 × 2 mm I.D., particle size: 2.6 μm, pore diameter: 150 Å; Thermo Fisher Scientific, ON, Canada) coupled to a Dionex Ultimate 3000 ultra-high performance liquid chromatography (UHPLC) system and a Q-Exactive Orbitrap high resolution accurate mass spectrometer (Thermo Fisher Scientific, ON, Canada) was used to resolve the lipids present in the complex standard mixture and *E. solidaginis* larvae fat. For chromatographic separation, solvent A composed of acetonitrile:water (60:40 v/v) while solvent B contained isopropanol:acetonitrile:water (90:10:1 v/v/v); with both solvents containing 10 mM ammonium formate and 0.1% formic acid. C30-RPLC separation was carried out at 30 °C (column oven temperature) with a flow rate of 0.2 mL/min, and 10 μL of the complex lipid extract suspended in chloroform was injected into the machine. The following gradient conditions were used: column equilibrium at 30% solvent B for 3 min; then solvent B increased to 50% over 3 min, then to 70% B in 3 min, followed by an increase to 90% B over 7 min, then to 99% B over 10 min and kept at 99% B for 4 min. The column was re-equilibrated to starting conditions (70% solvent A) for 5 min prior to each new injection. The following parameters were used for the Orbitrap mass spectrometer, sheath gas: 35, auxiliary gas: 15, ion spray voltage: 3.5 kV, capillary temperature: 300 °C; S-lens RF: 30 V; mass range: 100–1500 m/z; full scan mode at a resolution of 70,000 m/z; top-20 data de-pendent MS/MS at a resolution of 35,000 m/z and collision energy of 35 (arbitrary unit); injection time 35 min; isolation window: 1 m/z; automatic gain control target: 1e5. The Instrument was externally calibrated to 1 ppm using ESI negative and positive calibration solutions (ThermoScientific, MO, USA). Tune parameters were optimized using mixture of lipid standards (Avanti Polar Lipids, Alabama, USA) in positive ion modes. TG concentrations in *E. solidaginis* larvae fat are expressed on nanomol percent (nmol %) and microgram per gram fresh weight (μg/g FW) basis.

### Data processing

Xcalibur data acquisition and interpretation software (version 4.0, Thermo Fisher Scientific, Ontario, Canada) was used for the C30-RPLC-HRAM-MS/MS lipidomics data acquisition and processing. For the identification at a molecular level, the relative positions of medium and long-chain fatty acids (FA) in TG molecular species were assigned based on the general rules of the relative abundances of fragment ions in their MS/MS spectra. The rule was established based on common findings that the loss of fatty acid(s) at the *sn*-1 and *sn-*3 positions are observed at higher abundance than *sn*-2 fatty acid loss [28–30]. However, it was found to be an exception for TG containing short chain fatty acids. For example, in this study the loss of CH_3_COONH_4_ was observed at the lowest abundance in the MS/MS spectra of MAcDG standards although acetic acid was esterified to the *sn*-3 position. For relative quantitation, integrated peak areas and exact masses of TG molecular species were used to calculate the relative abundance (nmol %) of TG molecular species in *E. solidaginis* larvae fat. Furthermore, the absolute quantitation was conducted based on a limited number of available standards to estimate the concentration of TG molecular species in *E. solidaginis* larvae fat (μg/g FW). It was based on integrated peak areas of TG molecular species and standards curves generated from authenticated standards (Triglycerides mixtures- 17,811-1AMP) purchased from Supelco (Oakville Ontario, Canada) and MAcDG standards obtained from Chemforce Laboratories (Edmonton, Canada). The standard curves were generated using representative standards from each class due to the lack of standards for all individual molecular species, and thus not completely accounted for the matrix effects common in biological samples. However, it was found that the corresponding factor of lipid ions depended mainly on their headgroup, thus only one representative standard is required for each lipid class/subclass in absolute quantitation [26, 31].

### Statistical analysis

TG molecular species and subclass composition in *E. solidaginis* larvae fat were assessed using the descriptive statistics procedure in XLSTATS. One-way analysis of variance (ANOVA) was used to determine significant differences between the levels of TG molecular species and subclasses present in *E. solidaginis* larvae fat. Where differences were significant, the means were compared with Fisher’s Least Significant Difference (LSD), *α* = 0.05. XLSTAT (Premium version, Addinsoft, New York, USA) was used for statistical analysis.

## Results

### Analysis of MAcDG in complex lipid standard mixture using thin layer chromatography coupled to flame ionization detection (TLC-FID)

**Fig. 1:**
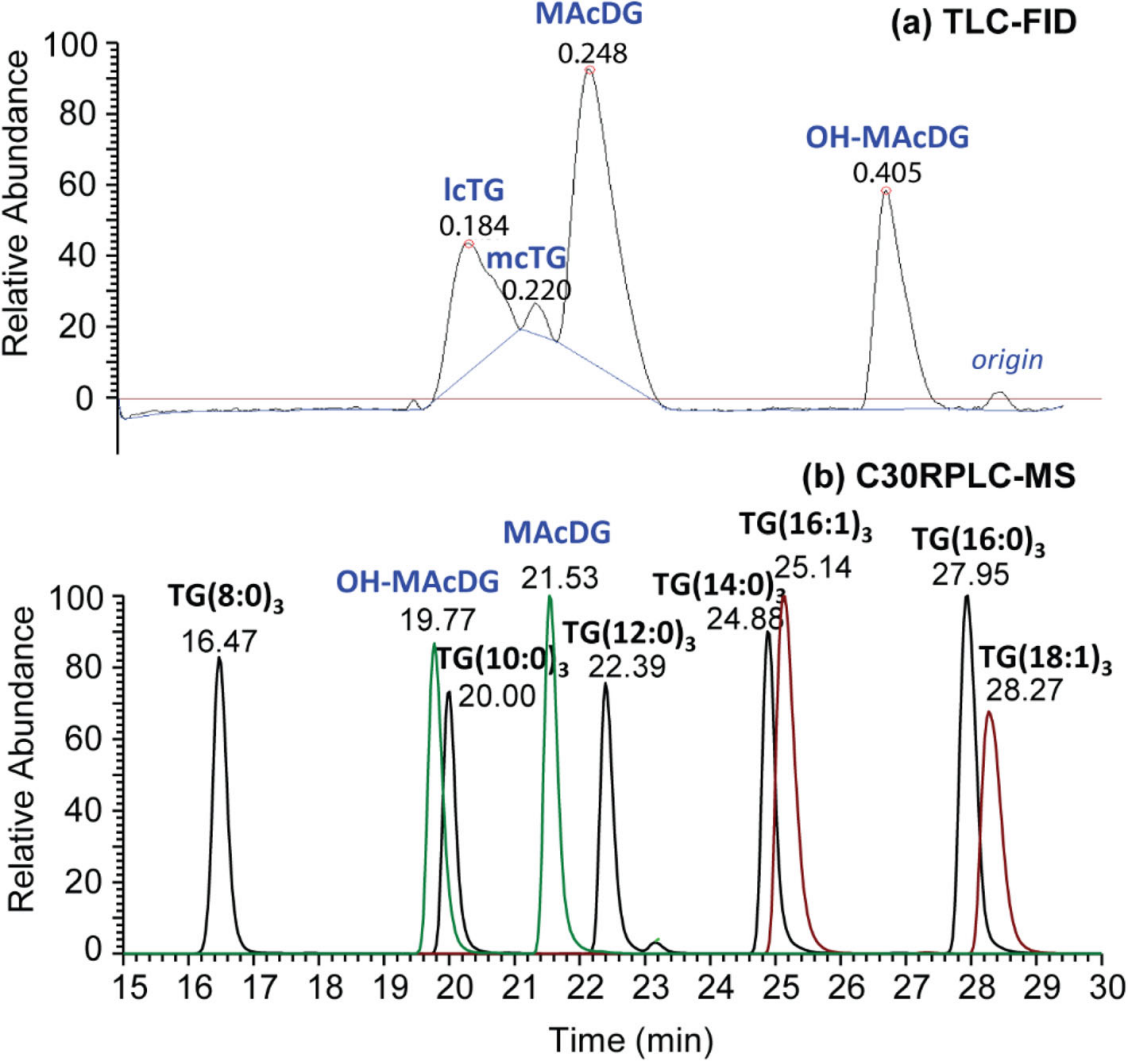
**a** TLC-FID separation of TG subclasses and **b** C30-RPLC-HESI-HRAM-MS/MS chromatograms in positive ion mode of the standard lipid mixture of monoacetyldiacylglycerols (MAcDG), hydroxylated monoacetyldiacylglycerols (OH-MAcDG) medium and long chain triglycerides (mcTG and lcTG)

One objective of this study was to demonstrate thin layer chromatography coupled with flame ionization detection (TLC-FID) as a simple analytical method for routine, accurate identification, and quantitation of MAcDG from other TG subclasses in complex biological samples. In the TLC-FID approach, a solvent system composed of benzene:chloroform:formic acid was utilized for chromatographic separation of neutral lipid classes on polar silica chromarods [20, 32]. It was observed that TG subclasses in the complex standard mixture separated into four major peaks based on retention times corresponding to OH-MAcDG (0.41 min), MAcDG (0.25 min), mcTG (0.22 min), and lcTG (0.18 min) subclasses (Fig. 1a). The retention times demonstrated the polarity of the different TG subclasses using this solvent system is as follows: OH-MAcDG< MAcDG< mcTG< lcTG (Fig. 1a).

### Analysis of MAcDG in complex lipid standard mixture using C30 reversed phase liquid chromatography coupled to heated electrospray ionization- high resolution accurate mass tandem mass spectrometry (C30-RPLC-HESI-HRAM- MS/MS)

**Fig. 2:**
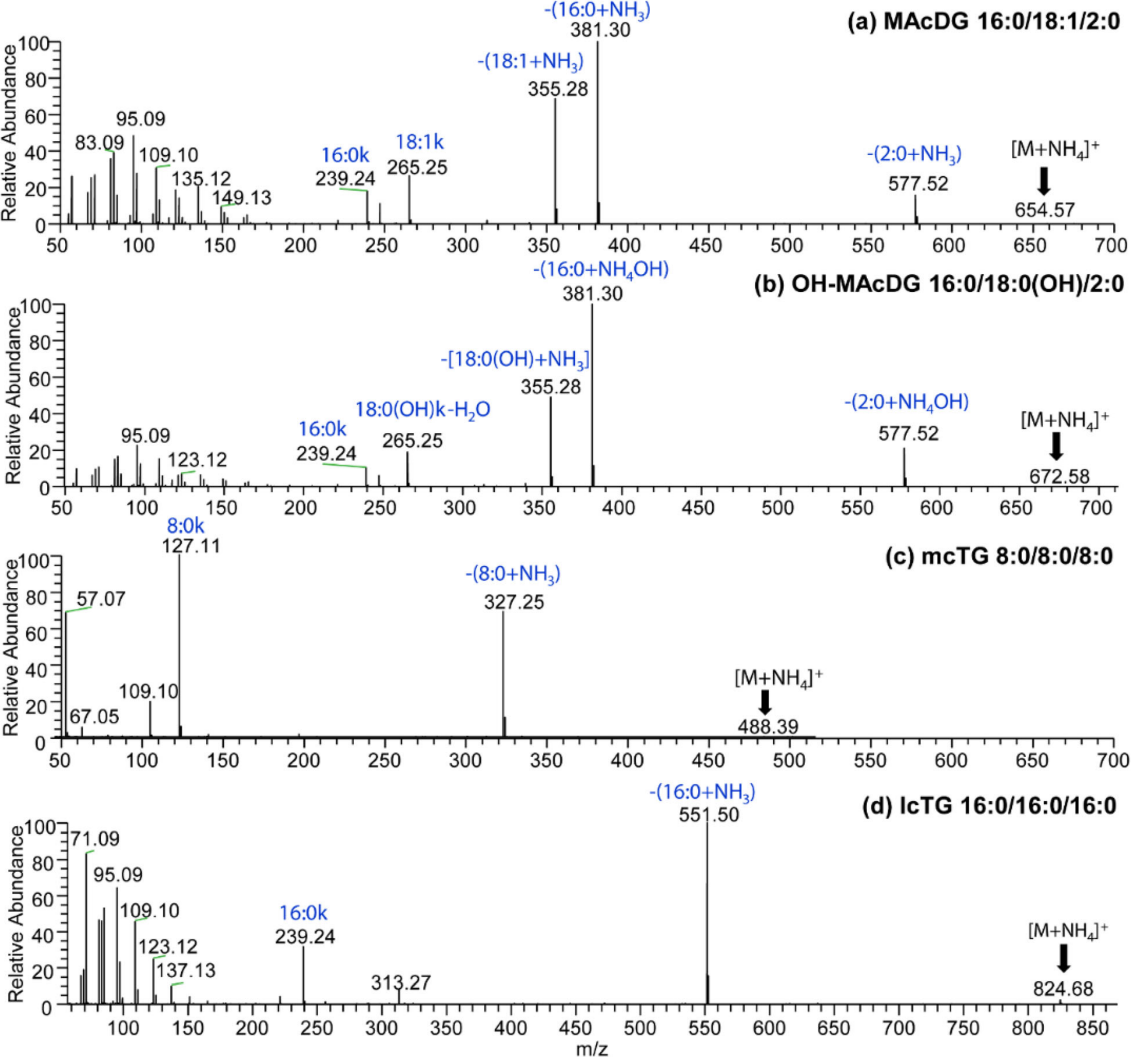
C30-RPLC-HESI-HRAM-MS/MS spectra showing fragmentation of precursor ions isolated at 1 Da selection window in positive ion mode for the lipid standards representing: **a**) MAcDG 16:0/18:1/2:0 (36:1) at *m/z* 654.57, **b**) OH-MAcDG 16:0/18:0(OH)/2:0 (OH-36:0) at *m/z* 672.58, **c**) mcTG 8:0/8:0/8:0 (24:0) at *m/z* 488.39 and, **d**) lcTG 16:0/16:0/16:0 at *m/z* 824.68. All TG subclasses are shown as ammonium adducts ([M + NH_4_]^+^

In order to investigate TG and MAcDG at molecular structural level, C30-reversed phase liquid chromatography coupled to high resolution accurate mass tandem mass spectrometry (C30-RPLC-HRAM-MS/MS) was used complimentary to TLC-FID for the simultaneous analysis of the fatty acids and molecular species composition of intact MAcDG and other TG subclasses in biological samples. To this end, a mobile phase consisting of solvent A (acetonitrile:H_2_O 60:40 v/v) and solvent B (isopropanol:acetonitrile:water 90:10:1 v/v/v) both containing ammonium formate buffer were used as the solvent system applied with C30-RPLC coupled with accurate mass tandem mass spectrometry optimized for separation and quantification of intact neutral lipid species. A clear resolution of TG molecular species including mcTG, lcTG MAcDG, and OH-MAcDG present in standard mixture was observed based on polarity, fatty acid composition, and fatty acid chain length ranging from TG(8:0)_3_ to TG(18:1)_3_, in positive ion mode (Fig. 1b). Resolution of non-acetylated TG species including mcTG: TG(8:0)_3_ occurred at 16.47 min, TG(10:0)_3_ at 20.00 min, and TG(12:0)_3_ at 22.39 min from lcTG: TG(14:0)_3_ at 24.88 min, TG(16:1)_3_ at 25.14 min, TG(16:0)_3_ at 27.95 min, and TG(18:1)_3_ at 28.27 min in the standards were achieved. These non-acylated TG molecular species were well separated from the acetylated TG including OH-MAcDG which occurred at 19.77 min and MAcDG at 21.53 min (Fig. 1b). The high resolving power of the Q-Exactive Orbitrap mass spectrometer allowed for accurate and unambiguous resolution and structural identification of TG molecular species including MAcDG and OH-MAcDG present in the standard mixture in positive ion mode (Fig. 2). The C30-RPLC-HRAM-MS/MS spectra of MAcDG and OH-MAcDG molecular species present in the standard mixture are shown in Fig. 2a-d. The proposed C30-RPLC-MS/MS method also allowed for resolution and identification of acylated TG (MAcDG and OH-MAcDG) and non-acylated TG (mcTG and lcTG) molecular species in the complex standard mixture. Ammonium formate buffer was used in the C30-RPLC method for column optimization. Therefore, all intact TG molecular species present in the standard mixture formed ammonium adducts [TG + NH_4_]^+^ under the C30-RPLC-HRAMS-MS/MS conditions used in this study. The high resolution mass spectrometry allowed for accurate quantification of each TG precursor ion species detected in MS scan based on their extracted ion [TG + NH_4_]^+^ peak area. The linear relationship between extracted *m/z* ion peak area and MAcDG concentration is observed even at low range, i.e., 0.1–10 μg/mL for the quantification of MAcDG (Fig. 6a-b).

### Application of C30-RPLC-HESI-HRAM-MS/MS for separation and identification of MAcDG molecular species in larvae *of E. solidaginis*

**Fig. 3:**
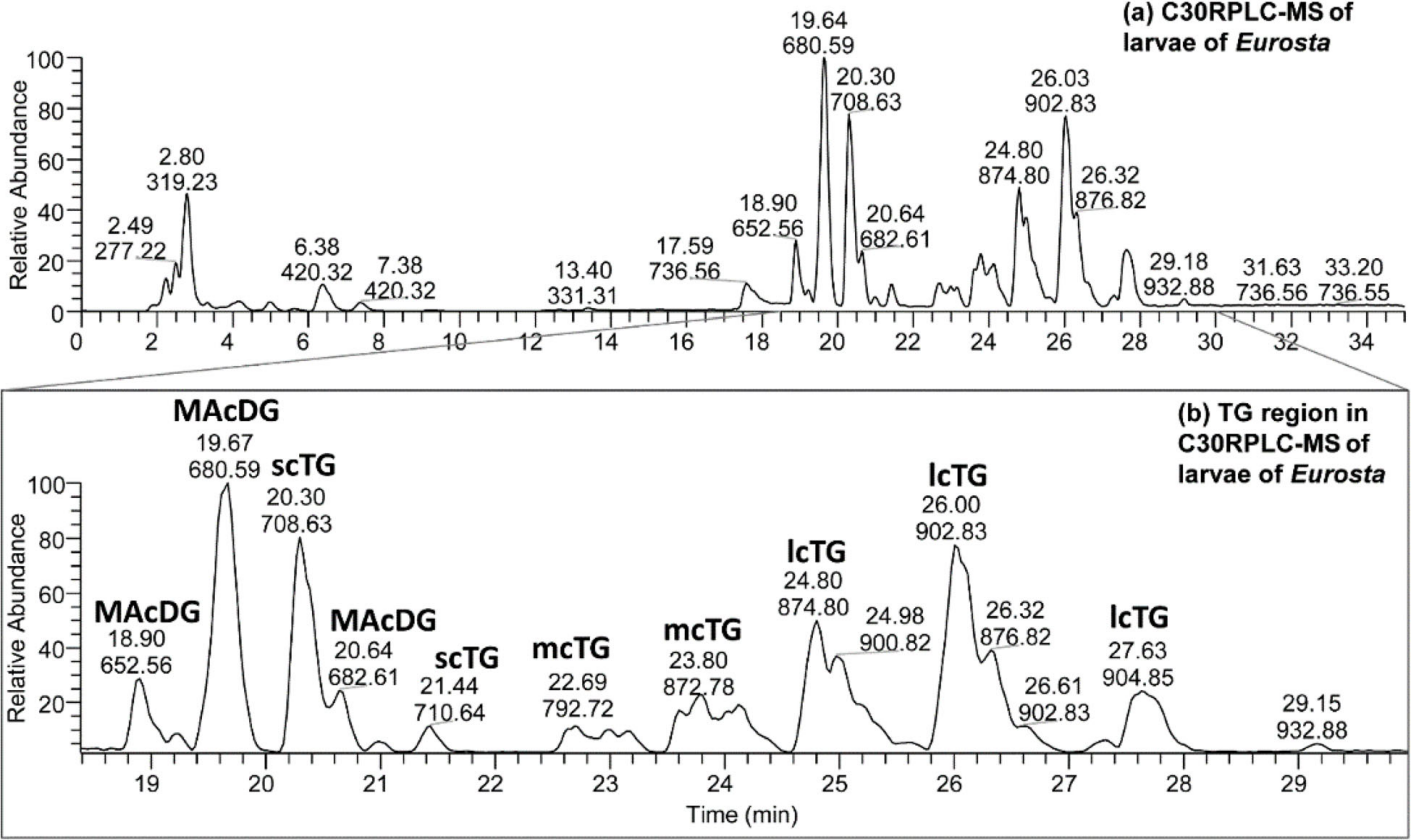
**a** C30-RPLC-HESI-HRAM-MS chromatogram in positive ion mode of complex lipid extracted from the larvae of *Eurosta solidaginis* species. **b** Extracted ion chromatogram of the neutral lipids region of the C30-RPLC-MS chromatogram showing some of the major MAcDG, scTG, mcTG and lcTG in the larvae lipid profile

**Fig. 4:**
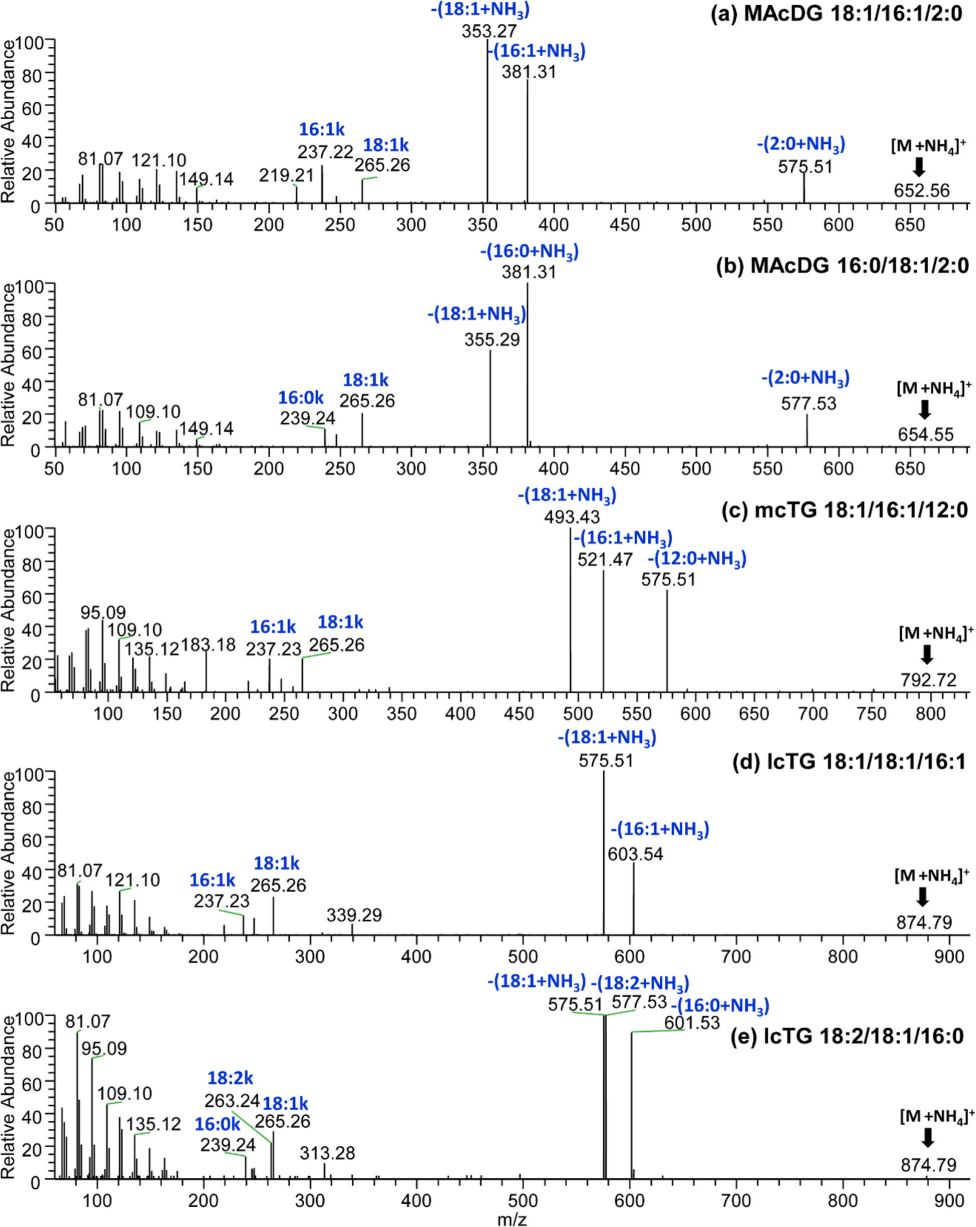
C30-RPLC-HESI-HRAM-MS/MS spectra showing fragmentation of precursor ions isolated at 1 Da selection window in positive ion mode for the major MAcDG species in *Eurosta solidaginis* species larvae corresponding to: **a**) MAcDG 18:1/16:1/2:0 (36:2) at *m/z* 652.56, **b**) MAcDG 16:0/18:1/2:0 (36:1) at *m/z* 654.55. **c)** mcTG 18:1/16:1/12:0 (46:2) *m/z* 792.72. Resolution of lcTG 52:3 isomers at *m/z* 874.79 representing: **d**) 18:1/18:1/16:1 and **e** 18:2/18:1/16:0

The proposed C30-RPLC-HRAM-MS/MS method was applied to separate MAcDG lipid species present in the lipidome of *E. solidaginis* larvae from other TG molecular species. It was observed that MAcDG eluted between 18.90 to 20.64 min followed by the other TG subclasses between 21.44 and 27.63 min under C30-RPLC conditions (Fig. 3a-b). Four main classes of TG molecular species were identified in the larvae of *E. solidaginis* including MAcDG, scTG, mcTG and lcTG (Fig. 3b). High resolution accurate mass tandem mass spectrometry (HRAM-MS/MS) was used to assign the molecular species composition of MAcDG, scTG, mcTG and lcTG species in *E. solidaginis* larvae lipidome as shown in Fig. 4a-e & Fig. S-1 – S-3.

**Fig. 5:**
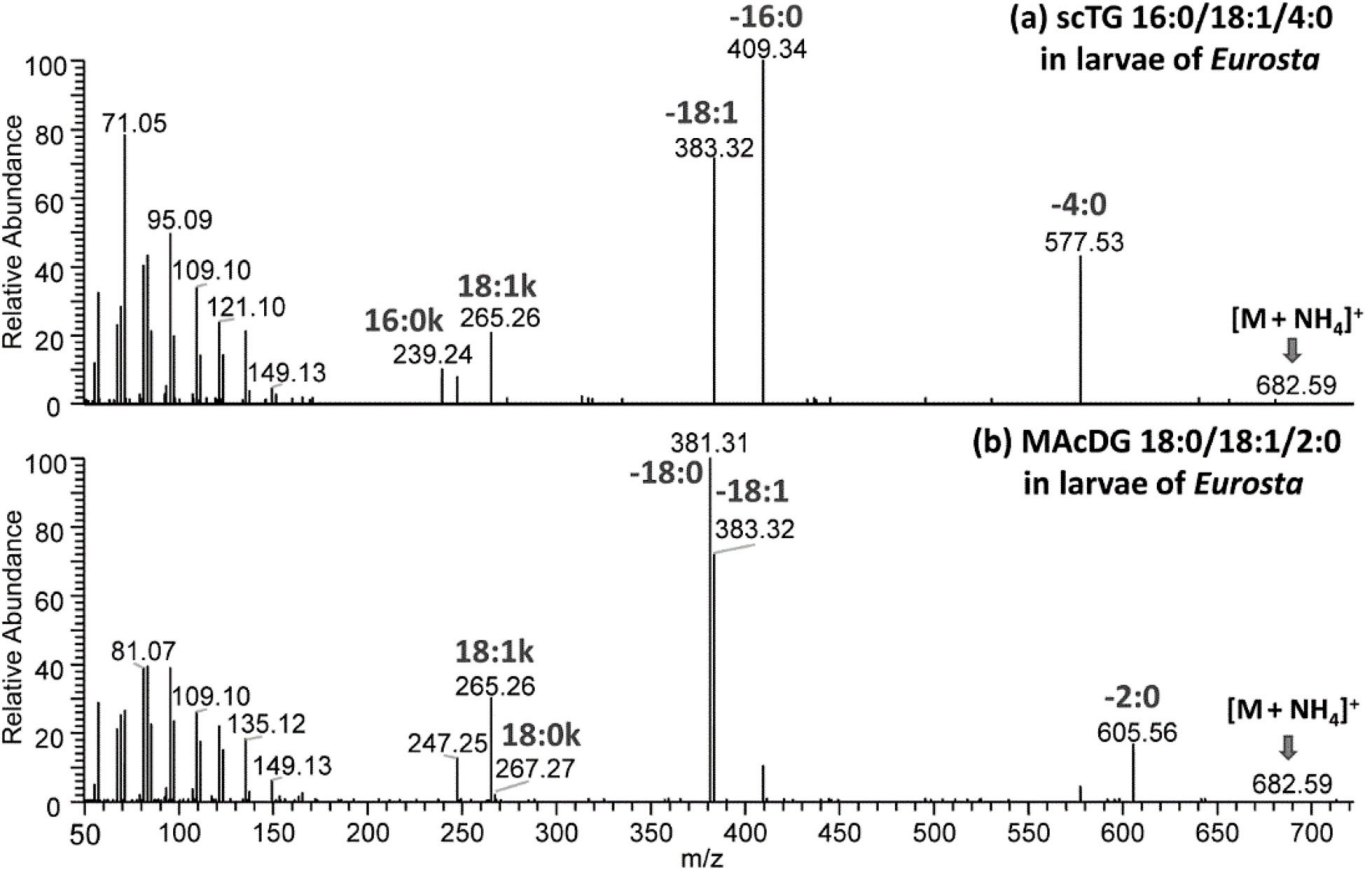
C30-RPLC-HESI-HRAM-MS/MS spectra showing fragmentation of precursor ions ions isolated at 1 Da selection window in positive ion mode for isomers of scTG and MAcDG in *Eurosta solidaginis* corresponding to: **a**) scTG 16:0/18:1/4:0 (38:1) at m/z 682.59 and **b**) MAcDG 18:0/18:1/2:0 (38:1) at *m/z* 682.59

**Fig. 6:**
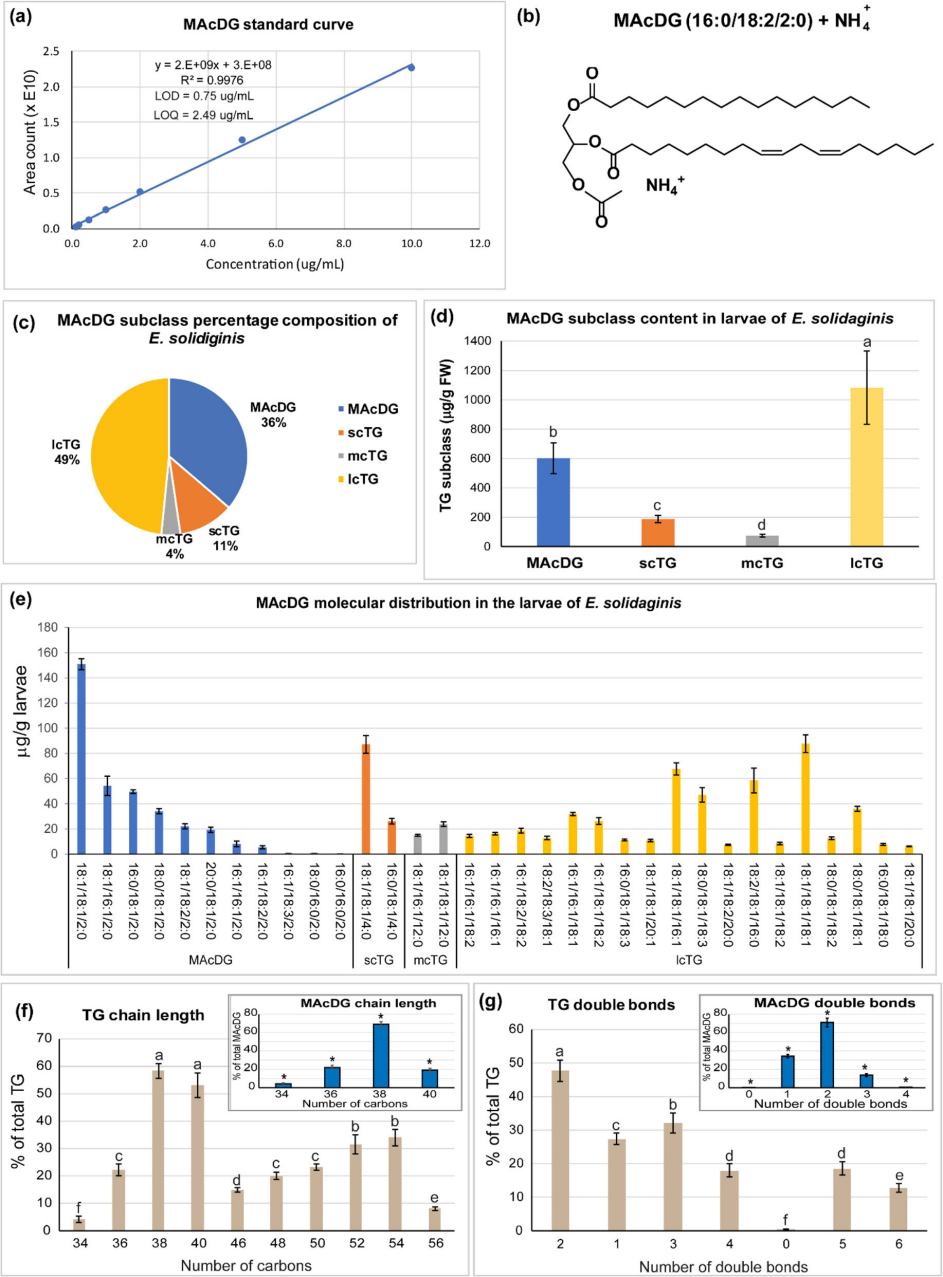
Quantitation and distribution of MAcDG and other TG subclasses present in the larvae of *E. solidaginis.* **a**) Standard curve used to calculate the amount of MAcDG in *E. solidaginis*, **b**) Representative structure of MAcDG, **c**) Percent composition, **d**) quantity, and **e**) molecular species distribution of MAcDG and other TG subclasses present in the larvae of *E. solidaginis.* **f**) Chain length as the number of carbons, **g**) and number of double bonds in the sum of the FA moieties in MAcDG present in *E. solidaginis*. Values represent means ± standard error (*N* = 7). Fisher LSD was used to separate the means at alpha = 0.05 following analysis using one-way ANOVA

Furthermore, it was possible to resolve isomers of MAcDG from that of other TG subclasses present in *E. solidaginis* larvae using the proposed method (Fig. 5a-b). The quantitation and distribution of MAcDG and other TG subclasses present in *E. solidaginis* larvae lipidome is shown in Fig. 6a-b. The neutral lipid content of *E. solidaginis* is enriched with MAcDG accounting for 36% of the TG composition, while lcTG accounted for 49% on mass percent basis (Fig. 6c-d). Herein it is further identified that the lipidome of *E. solidaginis* consisted of 11 and 4% scTG and mcTG respectively (Fig. 6c-d). At a molecular level, a total of 34 TG molecular species in *E. solidaginis,* which consisted of 12 MAcDG species, 2 mcTG species, 2 scTG species and 17 lcTG species were reported (Fig. 6e). The TG profile of *E. solidaginis* was enriched in PUFA containing TG consisting of 38 and 40 carbons, as well as two double bonds. Approximately, 48% of the non-acylated TG species consisted of 2 double bonds compared to 71% for MAcDG (Fig. 6f-g). Furthermore, MAcDG in *E. solidaginis* was composed of shorter carbon chains as compared to non-acylated TGs (C34-C40 vs C34-C56) with C38 predominating in both. However, MAcDG had higher levels of C38 carbon chains compared to other TG subclasses (69% vs 58%) as is shown in Fig. 6f-g*.*

## Discussion

The resolution of MAcDG and lcTG subclass in complex lipid standard mixture by TLC-FID presented in the current study is consistent with that reported by Marshall at al. (2014) for the analysis of *E. solidaginis* larvae fat [20]. The concentration range observed to provide excellent resolution and quantitation using TLC-FID is 100–1000 μg/mL (0.1 to 1 mg/mL). Below 100 μg/mL the peak size and appearance was not consistent, and above 1000 μg/mL the peaks shape appeared large and broad that can interfere with the resolution of MAcDG from the different TG subclasses. Furthermore, the response is linear between 100 and 1000 μg/mL for the quantification of MAcDG as well as other TG subclasses. The amount of MAcDG in a sample can be quantified by TLC-FID from the average peak area of MAcDG (7 chromarods) present in each sample using the linear regression equation (*y* = *mx* + *c*) and calculated as follows [17]:

$$ MAcDG\ \left(\mu g/g\kern0.5em sample\right)=\frac{\left(y-c\right)x\ V}{m\ x\ W} $$

where *y* = average peak area of MAcDG, *c* and *m* = intercept and slope of the regression line, respectively, *V* = total volume of lipid solution (mL), and *W* = weight of sample used (g).

The output presented in this paper demonstrate the ability of the method for simple, rapid, and sensitive quantification of OH-MAcDG, MAcDG, mcTG and lcTG in biological samples. This demonstrated that hydroxylated MAcDG (OH-MAcDG) was resolved from other lipid classes using TLC-FID and presented opportunities for purification and further evaluation of the hydroxylated version of MAcDG, which is a more polar version of MAcDG, as well as determine possible functions and applications. It is well recognised that the bioactivities and physical properties of MAcDG is distinctly different from that of other TG subclasses. How hydroxylation further influences these properties is unknown. The application of TLC-FID to resolve and quantify polar and neutral lipid classes in complex biological samples ranging from animal (egg yolk, chicken fat, lamb fat, milk), marine (lobster, krill oil, red porgy wild, greater weever, piper gurnard) plant (sesame seeds, canola gum, macadamia nuts, olive oil), and edible fungus (mushrooms) origins have been widely reported [19, 33–35]. These applications demonstrate the versatility of this analytical technique for routine, simple, efficient, accurate, and sensitive analysis of neutral lipids. MAcDG can now be included as a subclass in routine neutral lipid analysis in these sources using TLC-FID. The proposed TLC-FID method provides a simple, comparatively inexpensive, sensitive, and rapid method to separate, identify and quantitate TG subclasses classes including MAcDG and OH-MAcDG in biological samples. In particular, the unique biochemical composition of MAcDG in biological samples including *E. solidaginis* can confer novel uses and application in the food science field (considering insects are emerging sources of dietary proteins and health-promoting functional lipids), healthcare (treating sepsis, asthma, arthritis, cancers and tumors), and biofuel industry (potential additive to improve performance of biofuels in cold climates [26, 36]. These uses suggest possible applications for MAcDG for which rapid, relatively inexpensive, accurate and sensitive analytical methods for quantification will be essential. TLC-FID has been demonstrated in enabling the separation and analysis of MAcDG and OH-MAcDG subclasses across a range of biological samples.

Under the C30-RPLC-HRAM-MS/MS conditions used in present study, the presence of OH group in OH-MAcDG appears to have increased the relative polarity of MAcDG-OH thereby reducing its retention on the C30-RP column relative to MAcDG and lcTG species in the standard mixture [37]. As such MAcDG-OH eluted before MAcDG and lcTG species, as shown in Fig. 1b. Furthermore, separation of mcTG and lcTG molecular species present in the standard mixture was based on their fatty acid chain lengths [26]. The hydrophobic interactions between the stationary phase and hydrophobicity of fatty acyl chains (based on chain length, number and position of double bonds of fatty acids) were known to resolve neutral lipid species present in the standard mixture [38]. McTG molecular species have shorter fatty acyl chains compared to lcTG species which makes the former less hydrophobic compared to lcTG. As such, mcTG molecular species were less retained under C30-RPLC conditions compared to the more hydrophobic lcTG species [39].

The MS/MS spectrum of MAcDG 16:0/18:1/2:0 [M + NH_4_]^+^ at *m/z* 654.57 is shown in Fig. 2a. The diagnostic *sn-3* acetyl moiety at *m/z* 577.52 corresponds to the neutral loss of [CH_3_COO^−^NH_4_^+^] or 77 Da from *m/z* 654.57 [MAcDG 16:0/18:1/2:0 + NH_4_]^+^ molecular ion [40]. The composition and positions of fatty acids (FA) in TG molecular species including MAcDG and OH-MAcDG present in the standard mixture were identified based on the neutral loss of the fatty acid fragments as ammonium adducts, and presence of the fatty acid ketene ions [FA + H-H_2_O]^+^ in the MS/MS spectra [26, 41]. As such for MAcDG 16:0/18:1/2:0 (Fig. 2a), the fragment at *m/z* 355.28 correspond to the neutral loss of 18:1 + NH_3_ (*sn-2* FA) or 299 Da, while the fragment at *m/z* 381.30 correspond to the neutral loss of 16:0 + NH_3_ (*sn-1* FA) or 273 Da from *m/z* 654.57 [MAcDG 16:0/18:1/2:0 + NH_4_]^+^ ion [42]. Distinction between *sn-2* and *sn-1* fatty acids is based on the relative abundance of these two FA fragments, with relative abundance of *sn-2* FA fragment [18:1 + NH_3_] lower than that of *sn-1* FA fragment [16:0 + NH_3_], which is a trend typical of *sn-1* and *sn-2* FAs in TG [43]. Furthermore, fragment ions at *m/z* 239.24 and 265.25 in Fig. 2a were assigned to fatty acid ketene ions [16:0 + H-H_2_O]^+^ and [18:1 + H-H_2_O]^+^ corresponding to *sn-1* FA and *sn-2* FA, respectively. The distinction between *sn-2* and *sn-1* positions on TG molecular species including MAcDG and OH-MAcDG could also be identified by the relative abundance of these fatty acid ketene ions [26]. The relative abundance of [18:1 + H-H_2_O]^+^ ketene ion at *m/z* 265.26 corresponding to *sn-2* FA was higher than that of [16:0 + H-H_2_O]^+^ ion at *m/z* 239.24 for *sn-1* FA of MAcDG 16:0/18:1/2:0, which is a trend typical of *sn-1* and *sn-2* FA ketene ions in TG species (Fig. 2a). Thus, the trend based on relative abundance of *sn-1* and *sn-2* [FA + H-H2O]^+^ ketene ions is opposite to that observed for fragments formed by neutral loss of FAs from [MAcDG 16:0/18:1/2:0 + NH_4_]^+^ ion, and is consistent with the literature on determining TG molecular species [41, 42].

The proposed C30-RPLC-MS/MS method also allowed for resolution and identification of OH-MAcDG molecular species in the complex standard mixture. Structurally, while OH-MAcDG and MAcDG are distinguished from non-acetylated TG species including scTG, mcTG and lcTG by the presence of *sn-3* acetyl groups, OH-MAcDG differs from MAcDG by having one hydroxyl group on the *sn-2* fatty acid chain. These structural differences were seen in the mass spectra of MAcDG and OH-MAcDG molecular species present in the standard mixture under C30-RPLC-MS/MS conditions (Fig. 2a-b). For example, the MS/MS spectra of OH-MAcDG 16:0/18:0(OH)/2:0 *m/z* 672.58 is shown in Fig. 2b. The neutral loss of [CH_3_COO^−^NH_4_^+^] or 77 Da from [TG + NH_4_]^+^ ions is representative of MAcDG molecular species [40]. In contrast OH-MAcDG molecular species are distinguished from MAcDG by the neutral loss of [CH_3_COO^−^NH_4_^+^ + H_2_O] or 95 Da from [TG + NH_4_]^+^ ions [44]. Accordingly, the fragment at *m/z* 577.52 corresponding to neutral loss of [CH_3_COO^−^NH_4_^+^ + H_2_O] or 95 Da from *m/z* 672.58 [OH-MAcDG 16:0/18:0(OH)/2:0 + NH_4_]^+^ ion is diagnostic of the *sn-3* acetate moiety in OH-MAcDG 16:0/18:0(OH)/2:0 (Fig. 2b). Furthermore, relative abundances of fatty acid fragments arising from neutral loss from [TG + NH_4_]^+^ ions and fatty acid ketene ions were used to assign *sn-1* and *sn-2* fatty acid composition of OH-MAcDG species. Consistent with conventions in the literature for assigning the *sn*-*1* and *sn*-*2* fatty acids based on relative abundance [43], fragments at *m/z* 355.28 (18:0 *sn-2*) and *m/z* 381.30 (16:0 *sn-1*) correspond to neutral loss of [18:0 + NH_3_] and [16:0 + NH_3_] from [OH-MAcDG 16:0/18:0(OH)/2:0 + NH_4_]^+^ ion, respectively (Fig. 2b). As previously alluded to, the relative abundance of fragment corresponding to *sn-2* FA is lower compared to *sn-1* FA fragment [43]. In a similar fashion, the fragments at *m/z* 239.24 and *m/z* 265.25 were diagnostic of fatty acid ketene ions [16:0 + H-H_2_O]^+^ and [18:0 + H-H_2_O]^+^ respectively, with the relative abundance of the *sn-2* FA ketene ion higher than the *sn-1* FA ketene ion (Fig. 2b). In summary, neutral loss of 77 Da and 95 Da from [TG + NH_4_]^+^ ions was used to distinguish between MAcDG and OH-MAcDG molecular species present in the standard mixture.

In contrast, non-acetylated TG including scTG, mcTG and lcTG present in the standard mixture did not form fragments corresponding to neutral loss of 77 Da and 95 Da from [TG + NH_4_]^+^ ions under C30-RPLC-HRAM-MS/MS conditions, which provided a basis for their identification relative to acylated TG (MAcDG and OH-MAcDG). For example, the MS/MS spectra of mcTG 8:0/8:0/8:0 *m/z* 488.39 is shown in Fig. 2c. The neutral loss of 161 Da is representative of C8:0 FA loss from *m/z* 488.39 [mcTG 8:0/18:0/8:0 + NH_4_]^+^ ion, which corresponds to the fragment at *m/z* 327.25 (Fig. 2c), while the fragment at *m/z* 127.11 corresponds to the fatty acid ketene ion [8:0 + H-H_2_O]^+^ at *sn-1*, *sn-2* and *sn-3* positions of mcTG 8:0/8:0/8:0 [26]. The same approach was applicable for structural elucidation of lcTG molecular species present in the standard mixture. Accordingly, the *m/z* 551.50 fragment in Fig. 2d corresponds to the neutral loss of FA 16:0 (273 Da) from *m/z* 824.68 [lcTG 16:0/16:0/16:0 + NH_4_]^+^ ion, while fatty acid ketene ion [16:0 + H-H_2_O]^+^ was at *m/z* 127.11 [26, 28]. Application of C30-RPLC-MS/MS to identify and quantify neutral lipid molecular species in biological samples have been reported in the literature. Narvaez et al. (2016) applied a RPLC-MS/MS method in positive ion mode to resolve and analyze neutral lipids including TG in rat liver and rat plasma lipid extracts [22]. A similar application has been used for analysis of TG composition of Calu-3 cells, rat blood and human skin tissues [45]. However, none of these techniques have included the analysis of MAcDG and OH-MAcDG. The method reported herein utilizing a C30-RPLC-HRAM-MS/MS technique to analyze neutral lipids including TG was successful in detecting MAcDG in wild cervid meats (moose and caribou) [46]. Taken together, the proposed C30-RPLC-HRAM-MS/MS method allows for accurate detection and quantification of TG molecular species including OH-MAcDG and MAcDG with good resolution, sensitivity, and throughput during routine lipidomics.

TG subclass and molecular species composition in *E. solidaginis* is of interest due to its role as a model system in cold stress tolerance. In this model system, MAcDG have been demonstrated to provide cryoprotection during exposure to low temperature stress [20]. This work has also led to subsequent studies recently on the potential of producing specialized oil seed crops with superior levels of MAcDG for applications in the production of biofuels specific for cold temperatures or northern climates [15, 47]. For example, Liu et al. (2015) reported that EaDAcT genetically modified camelina and soybean accumulated MAcDG at up to 70 mol% of the total seed oil produced by the resultant crops. A similar strategy of genetic modification increased MAcDG content to 85 mol% in field-grown transgenic camelina [15]. Furthermore, recent interests in insects as a potential source of proteins and functional ingredients for food and animal feed to improve population health and wellbeing has gained recognition, for which the larvae of *E. solidaginis* could be a promising food source [48, 49]. TG in *E. solidaginis* larvae lipidome have been reported to contain about 36 mol% MAcDG compared to long chain TG [20]. However, no report was done on the molecular species composition of MAcDG in *E. solidaginis.* MAcDG is associated with several health benefits in human and these include treating sepsis [10], tumor growth and cancers [11], rheumatoid arthritis [12] and asthma [13].

In the *E. solidaginis* chromatogram, a clear separation of these TD subclasses was observed based on hydrophobicity, chain lengths and molecular weight as follows: MAcDG< scTG< mcTG< lcTG (Fig. 3b). No OH-MAcDG specie was observed in *E. solidaginis* lipidome, which is in line with the literature [20]. The C30-RPLC separated the more polar MAcDG species from non-acetylated TG species (scTG, mcTG and lcTG), which allowed for facile mass spectrometric identification and quantification (Fig. 3b). The MS/MS spectra of MAcDG 18:1/16:1/2:0 [M + H]^+^ at *m/z* 652.56 is shown in Fig. 4a. The fragment at *m/z* 575.51 corresponds to the neutral loss of 77 Da [CH_3_COO^−^NH_4_^+^] from *m/z* 652.56 [MAcDG 18:1/16:1/2:0 + NH_4_]^+^ ion. This neutral loss 77 Da is representative of the *sn-3* acetyl moiety [CH_3_COO^−^NH_4_^+^] characteristic of all MAcDG species. The two fatty acid fragments were at *m/z* 381.31 (16:1 *sn-2*) and *m/z* 353.27 (18:1 *sn-1*) corresponding to neutral loss of [16:1 + NH_3_] and [18:1 + NH_3_] from [MAcDG 18:1/16:1/2:0 + NH_4_]^+^ ion, respectively (Fig. 4a). Furthermore, corroboration of the fatty acid composition of MAcDG 18:1/16:1/2:0 was also based on the diagnostic fatty acid ketene ions at *m/z* 237.22 (*sn-2* FA) and *m/z* 265.26 (*sn-1* FA) which correspond to [16:1 + H-H_2_O]^+^ and [18:1 + H-H_2_O]^+^ ions, respectively (Fig. 4a). Assignment of *sn-1* FA and *sn-2* FA of MAcDG 18:1/16:1/2:0 was made based on the relative abundance of the neutral loss FA fragments and fatty acid ketene ions as elaborated previously in the discussion. A similar approach was applied to elucidate the structures of other MAcDG species detected in *E. solidaginis* larvae lipidome (Fig. 4b & Fig. S-1 – S-3). Significantly, the proposed C30-RPLC-HRAM-MS/MS method provided facile resolution of MAcDG and TG molecular species present as isomers in *E. solidaginis* larvae lipidome. For example, the MS/MS spectra of MAcDG species present in *E. solidaginis* larvae were distinguished from scTG, mcTG and lcTG species by the neutral loss of [CH_3_COO^−^NH_4_^+^] or 77 Da from [TG + NH_4_]^+^ ion, which is diagnostic of the *sn-3* acetyl moiety of all MAcDG species (Fig. 4a-e). Using the approach based on the relative abundances of neutral loss of the fatty acid fragments and fatty acid ketene ions explained above, as well as in the literature [28], the two isomers at *m/z* 874.79 [TG 52:3 + NH_4_]^+^ reported in Fig. 4d-e were assigned as lcTG 18:1/18:1/16:1 and lcTG 18:2/18:1/16:0 molecular species respectively.

The utility of the proposed of C30-RPLC-MS/MS for resolving isomers of MAcDG from that of other TG subclasses present in *E. solidaginis* larvae was also demonstrated. For example, MAcDG 18:0/18:1/2:0 [M + H]^+^ *m/z* 682.59 eluted at 20.64 min under C30-RPLC conditions (Fig. 5b) is an isomer of scTG 16:0/18:0/4:0 [M + H]^+^ *m/z* 682.59 which eluted at 21.44 min (Fig. 5a). These isomers were easily resolved based on their MS/MS fragmentation patterns (Fig. 5a-b). For scTG 16:0/18:1/4:0, the three fatty acid composition were assigned as follows: the fragments at *m/z* 577.53 is diagnostic of 4:0 fatty acid at *sn-3*, *m/z* 409.34 for 16:0 at sn*-1* and *m/z* 383.32 for 18:1 at the *sn-2* positions of the glycerol moiety. This correspond to the neutral loss of [4:0 + NH_3_], [16:0 + NH_3_] and [18:1 + NH_3_] from *m/z* 682.59 [scTG 16:0/18:2/2:0 + NH_4_]^+^ ion, respectively (Fig. 5a). Assignment of the *sn-1*, *sn-2* and *sn-3* FAs of scTG 16:0/18:0/4:0 was based on the relative abundance of these fragments with *sn-1* FA > *sn-2* FA > *sn-3* FA, a trend typical of TG species [26, 28]. Furthermore, diagnostic fatty acid ketene ions at *m/z* 239.24 and *m/z* 265.26 correspond to [16:0 + H-H_2_O]^+^ and [18:1 + H-H_2_O]^+^, which is representative of *sn-1* FA and *sn-2* FA respectively (Fig. 5a). In contrast, the structure of MAcDG 18:0/18:1/2:0 [M + H]^+^ *m/z* 682.59 in Fig. 5b was distinguished from scTG 16:0/18:1/4:0 by the fragment at *m/z* 605.56 which correspond to neutral loss of 77 Da from *m/z* 682.59 [MAcDG 18:0/18:1/2:0 + NH_4_]^+^ ion. Assignment of the *sn-1* and *sn-2* FAs in MAcDG 18:0/18:1/2:0 was based on the relative abundance of the fragments associated with the neutral loss of each fatty acid and the fatty acid ketene ions (Fig. 5b). A similar approach was used to assign all TG and MAcDG molecular species including isomers present in *E. solidaginis* (Fig. 4-5 & Fig. S1-S2). This work shows the advantage of C30-RPLC-HESI-HRAM-MS/MS as a superior platform for resolving isomers of MAcDG from other TG subclasses in biological samples.

### Application of C30-RPLC-HESI-HRAM-MS/MS for separation and identification of MAcDG molecular species in larvae *of E. solidaginis* and comparisons with the literature

**Fig. 7:**
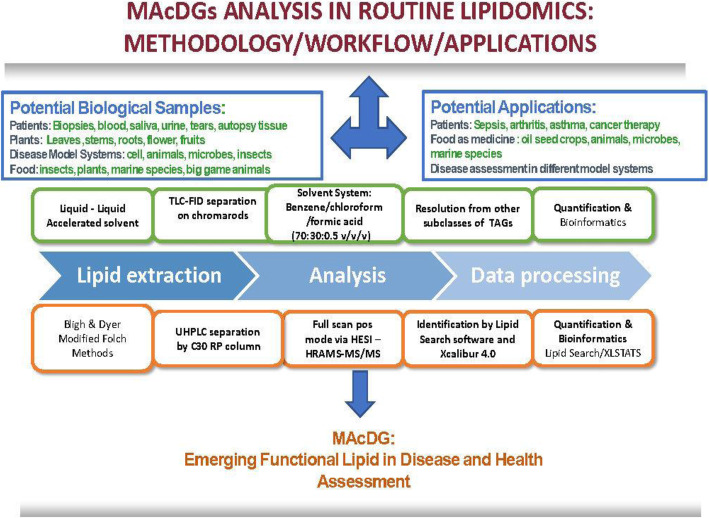
Flow diagram showing potential applications of the proposed multimodal approach for analysis of monoacetyldiacylglycerides in food ingredients, patients, health, and disease assessments during routine lipidomics. TAGs = triglycerides

TG subclass and molecular species composition in *E. solidaginis* is of interest due to its role as a model system in cold stress tolerance. In this model system, MAcDG have been demonstrated to provide cryoprotection during exposure to low temperature stress [20]. This work has also led to subsequent studies recently on the potential of producing specialized oil seed crops with superior levels of MAcDG for applications in the production of biofuels specific for cold temperatures or northern climates [15, 47]. For example, Liu et al. (2015) reported that EaDAcT genetically modified camelina and soybean accumulated MAcDG at up to 70 mol% of the total seed oil produced by the resultant crops. A similar strategy of genetic modification increased MAcDG content to 85 mol% in field-grown transgenic camelina [15]. Furthermore, recent interests in insects as a potential source of proteins and functional ingredients for food and animal feed to improve population health and wellbeing has gained recognition, for which the larvae of *E. solidaginis* could be a promising food source [48, 49]. TG in *E. solidaginis* larvae lipidome have been reported to contain about 36 mol% MAcDG compared to long chain TG [20]. However, no report was done on the molecular species composition of MAcDG in *E. solidaginis.* MAcDG is associated with several health benefits in human and these include treating sepsis [10], tumor growth and cancers [11], rheumatoid arthritis [12] and asthma [13]. The distribution of MAcDG and TG subclasses in *E. solidaginis* presented in the current work were consistent with those presented by Marshall et al. (2014) who demonstrated that the level of lcTG and MAcDG in *E. solidaginis* was 36 and 29% respectively, and varied with seasons under cold temperature stress following analysis using TLC-FID [20]. The output from this method shows for the first time the molecular species composition of MAcDG compared with other TG subclasses in *E. solidaginis.* This is important considering *E. solidaginis* can survive at cold temperatures as low as − 80 °C and is a well recognised model system for studying cold temperature stress in different biological systems. MAcDG has been previously reported to confer cryoprotective properties to *E. solidaginis* during temperature stress survival. Information on the molecular species composition of MAcDG will allow further work by researchers in the scientific community to improve the understanding of the mechanisms associated with MAcDG metabolism during cold temperature stress acclimation, therapies associated with sepsis, asthma, arthritis in patients (Fig. 7). Furthermore, insect larvae are an emerging source of high-quality dietary lipids. This information could be useful in the evaluation of *E. solidaginis* as a source of dietary MAcDG or functional lipids (Fig. 7). In summary, the proposed C30-RPLC-HRAM-MS/MS method appears to be a suitable approach for the analysis of MAcDG and other TG subclasses, molecular species, isomers, and fatty acid composition in *E. solidaginis* lipidome, that could also be applied for routine lipidomics analysis of other biological samples.

### Strength and limitations of the multimodal approach using high resolution C30-RPLC-HESI-HRAM-MS/MS and TLC-FID for routine MAcDG analysis

#### Strengths

The multimodal approach proposed in this paper is advantageous compared to other approaches in the literature in that TLC-FID facilitate quick, cost effective, simple resolution of MAcDG from other neutral lipids including subclasses of TG, (mcTG, scTG, lcTG), as well as polar lipids using silica rods and a non-polar solvent system. The flame ionization detection (FID) facilitates rapid, cost effective quantitation of total MAcDG in the sample. This approach demonstrated for the first time the resolution of hydroxylated MAcDG from other TG subclasses. Applying C30-RPLC-HESI-HRAM-MS/MS allow determination of the molecular species composition including the fatty acids composition of MAcDG in biological samples. Most lipidomics analysis currently use C18 RPLC columns to resolve TG and other neutral lipids during routine lipidomics (Fig. 7). We used C30 RPLC instead, due to the superior resolving power of C30 stationary phase in separating TG and other lipid isomers. When combined with accurate mass tandem mass spectrometry, this is a powerful platform to resolve MAcDG molecular species, fatty acids and isomers including positional isomers as demonstrated in this paper. This is very important because MAcDG was not typically analyzed as a component of routine or global lipidomics. Generally, a targeted approach was employed that often involved pre-concentration steps.

#### Limitations

Currently, there is no available commercial MAcDG standard for use in the scientific community. This is a major drawback in the method particularly during extraction and quantification. Having a unique molecular version of MAcDG would allow analysts to spike their test samples to better assess recovery, during extraction, and perform more accurate and precise quantification. The hydroxylated version of MAcDG presented in this paper is currently being explored as a suitable internal standard to fill this purpose. Furthermore, MAcDG is required for use of this method during lipidomics, particularly when using the TLC FID method. This means that analytical labs will need to purify or fractionate extracts of samples from *E. solidaginis*, transgenic camelina or soybeans known to have up to 70 mol% MAcDG [15] for use as pure standards in the analysis. Small quantities will be required for the LC-MS based analysis, but larger quantities (microgram quantities) will be required for the TLC-FID based analysis reported in this study. This is another limitation of the proposed method that could be an impediment to routine applications and adoption in lipidomics laboratories. We hope by highlighting these limitations and benefits that this will provide an impetus for other scientists in the scientific community to devise improved strategies using the information presented in this paper as a foundation to improve or enhance this method. Currently, work in our research group is showing MAcDG to be more common in biological samples than is currently appreciated in the scientific literature. Many times, they are incorrectly analyzed as different molecular species of short chain TG.

## Conclusion

In this work, TLC-FID and C30-RPLC-HESI-HRAM-MS/MS methods were investigated as a multimodal approach for the analysis of MAcDG, OH-MAcDG, scTG, mcTG and lcTG during routine lipidomics analysis. TLC-FID separated TG lipids into subclasses based on polarity, while C30 RPLC separated TG lipids based on chain length, polarity, and fatty acid composition. In this paper, the successful application of the C30-RPLC-HESI-HRAM-MS/MS method to separate and identify MAcDG, scTG, mcTG, and lcTG lipid subclasses was demonstrated. In addition, resolution of molecular species and isomers in *E. solidaginis* was present as an example of the suitability of the method for routine lipidomics analysis of biological samples. Furthermore, C30-RPLC-HESI-HRAM-MS/MS provided excellent intra-class resolution of TG subclasses and isomers with different fatty acid compositions. This method would increase the potential of including MAcDG in routine lipidomics of biological samples which would have broad interests and applications in the scientific community.

## Supplementary Information

**Additional file 1.** A multimodal analytical method to simultaneously determine monoacetyldiacylglycerols, medium and long chain triglycerides in biological samples.

## Data Availability

The data (raw and processed) used in the current study will be available from the corresponding author on reasonable request.

## Abbreviations

MAcDG: Monoacetyldiglycerides
FA: fatty acids
scTG: Short chain triglycerides
mcTG: Medium chain triglycerides
TG: Triglycerides
lcTG: Long chain triglycerides
DHA-ω3: Docosahexaenoic acid
EPA-ω3: Eicosapentaenoic acid
ALA-ω3: Linolenic acid
ARA-ω6: Arachidonic acid
LA-ω6: Linoleic acid
TLC-FID: Thin layer chromatography coupled with flame ionization detection
GC-FID: Gas chromatography coupled to flame ionization detection
GC-MS: Gas chromatography coupled to mass spectrometry
LC-HESI-MS/MS: Liquid chromatography-heated electrospray ionization tandem mass spectrometry
C30-RPLC-MS/MS: C30-reversed phase liquid chromatography-tandem mass spectrometry
OH-MAcDG: Hydroxylated MAcDG
LSD: Fisher’s Least Significant Difference
HRAM-MS/MS: High resolution accurate mass tandem mass spectrometry
[FA + H-H_2_O]^+^: Fatty acid ketene ions
