# Supplementary material for: A multimodal analytical method to simultaneously determine monoacetyldiacylglycerols, medium and long chain triglycerides in biological samples during routine lipidomics
Source: Lipids Health Dis. 2022 May 10;21:42. doi: 10.1186/s12944-022-01650-w (PMC9092795; doi:10.1186/s12944-022-01650-w)
Supplement: Supplementary file 1 — Additional file 1. A multimodal analytical method to simultaneously determine monoacetyldiacylglycerols, medium and long chain triglycerides in biological samples. [file 12944_2022_1650_MOESM1_ESM.docx]

**Supplementary**

**For**

**A multimodal analytical method to simultaneously determine monoacetyldiacylglycerols, medium and long chain triglycerides in biological samples.**

Charles F. Manful*, Thu H. Pham, Heather Spicer, Raymond H. Thomas*



 **Fig. S-1** C30-RPLC-HESI-HRAMS/MS spectra showing precursor ions in positive mode for the minor MAcDG species in *Eurosta solidaginis* corresponding to: **a)** MAcDG 16:1/16:1/2:0 (34:2) at *m/z* 624.53. **b)** MAcDG 16:0/16:1/2:0 (34:1) at *m/z* 626.49. **c)** MAcDG 16:0/16:0/2:0 (34:0) at *m/z* 628.50. All TG subclasses including MAcDG are shown as ammonium adducts ([M+NH_4_]^+^.





**Fig. S-2** C30-RPLC-HESI-HRAMS/MS spectra showing precursor ions in positive mode for the minor MAcDG species in *Eurosta solidaginis* corresponding to: **a)** MAcDG 16:1/18:3/2:0 (36:4) at *m/z* 648.49. **b)** MAcDG 16:1/18:2/2:0 (36:3) at *m/z* 650.54. **c)** MAcDG 16:0/18:3/2:0 (36:3) at *m/z* 650.54. **d)** MAcDG 18:1/16:1/2:0 (36:2) at *m/z* 652.55. **e)** MAcDG 16:0/18:1/2:0 (36:1) at *m/z* 654.45. **f)** MAcDG 18:0/16:0/2:0 (36:0) at *m/z* 656.50. All TG subclasses including MAcDG are shown as ammonium adducts ([M+NH_4_]^+^.





**Fig. S-3** C30-RPLC-HESI-HRAMS/MS spectra showing isomeric precursor ions in positive mode for the lipid species in *Eurosta solidaginis* representing: **a)** scTG 18:1/18:1/4:0 (40:2) at *m/z* 708.52. **b)** scTG 18:0/18:1/4:0 (40:1) and **c)** MAcDG 20:0/18:1/2:0 (40:1) at *m/z* 710.64 isomers. All TG subclasses including MAcDG are shown as ammonium adducts ([M+NH_4_]^+^.
